# PgRNA closely correlates to cytokine profile in HBeAg-positive pregnant women undergoing prophylactic antiviral intervention

**DOI:** 10.3389/fimmu.2024.1511855

**Published:** 2024-12-11

**Authors:** Qiao Tang, Chunrui Wang, Hu Li, Zhiwei Chen, Xiaoqing Liu, Yunling Xue, Yue Qiu, Yi Zeng, Peng Hu

**Affiliations:** ^1^ The Second Affiliated Hospital of Chongqing Medical University, Chongqing, China; ^2^ Department of Infectious Diseases, The First Affiliated Hospital of Chongqing Medical University, Chongqing, China; ^3^ Institute for Viral Hepatitis, The Key Laboratory of Molecular Biology for Infectious Diseases, Chinese Ministry of Education, Chongqing Medical University, Chongqing, China

**Keywords:** cytokine, immune, PgRNA, HBcrAg, pregnancy, hepatitis B virus, chronic hepatitis B, ALT flare

## Abstract

**Background:**

Previous studies primarily focused on the effects of ALT and virology, but there is a lack of research on the correlations of HBcrAg and pgRNA, two novel virologic markers, with immunological parameters in pregnant women with CHB undergoing prophylactic antiviral intervention.

**Methods:**

We conducted a retrospective cohort study involving 28 HBeAg-positive pregnant women with CHB undergoing prophylactic antiviral intervention. Clinical data, virological markers (HBV DNA, HBsAg, HBeAg, HBcrAg and pgRNA) and 28 cytokines were detected at three time points: 24-28 weeks gestation (before prophylactic antiviral intervention), near birth and within 3 months postpartum.

**Results:**

PgRNA was moderately (correlation coefficient between 0.4 and 0.6) positively correlated with Th1-type cytokines (IFN-γ, IL12p70, IL2, and TNF-α), Th17-type cytokines (IL21), Th2-type cytokines (IL10, IL4, and IL5), and cytokines regulating cell proliferation and differentiation (CTLA4, IL15, IL23, and TGF-β1) and moderately negatively correlated with EGF (correlation coefficient = -0.4), while ALT, HBV-DNA, HBsAg and HBcrAg were insignificantly correlated with cytokines at 24-28 weeks of gestation. Most cytokines tended to be elevated, with statistically significant increases observed only for the chemokines IP10 and MCP-1 during pregnancy. Most cytokines were significantly increased in postpartum women with virologic rebound after treatment discontinuation postpartum, but no significant change in the Th1/Th2 ratio. Changes in virologic markers were significantly correlated with cytokines. Immune activation was more pronounced in postpartum women who developed ALT flare compared to who did not, with Th1-type cytokines (especially IL12p40) and chemokines being main differential cytokines.

**Conclusion:**

PgRNA was more closely correlated with cytokine profiles, and postpartum ALT flare may be the result of the interaction between Th1-type cytokines and chemokines.

## Highlights

- We first comprehensively characterized the dynamics of cytokine profile, as well as HBcrAg and pgRNA profiles, and their correlations in HBeAg-positive pregnant women with CHB undergoing prophylactic antiviral intervention.
- We found a closer correlation between pgRNA and cytokine profiles, compared to HBcrAg and traditional virologic markers HBsAg and HBV DNA.
- The immune status was relatively stable during pregnancy and showed immune activation postpartum, and changes in virologic markers were closely correlated with cytokines changes.
- Immune activation was more pronounced in patients developed ALT flare after discontinuing treatment postpartum, and postpartum ALT flare may be the result of the interactions between Th1-type cytokines and chemokines, and IL12p40 may be the most crucial cytokine.

## 1 Introduction

Mother-to-child transmission remains a major route of hepatitis B virus (HBV) transmission in China (1). Current guidelines recommend prophylactic antiviral intervention for pregnant women with chronic hepatitis B (CHB) who are HBeAg-positive or high viral loads (2–4). Previous studies primarily focused on the effects on ALT and virology, with limited exploration of immunology, especially cytokine profiles, following prophylactic antiviral intervention in pregnant women with CHB (5–7). Previous studies suggest that hormonal fluctuations during pregnancy influence the immune status (8, 9). Cytokines, as key mediators of immune responses, play a crucial role in HBV infection and life cycle (10). Therefore, investigating cytokine profiles may provide valuable insights into the immune status of pregnant women with CHB undergoing prophylactic antiviral intervention.

HBcrAg and pgRNA are novel virologic markers, and previous studies on these two markers have mainly focused on non-pregnant CHB patients (11, 12). Our previous studies explored HBcrAg and pgRNA profiles in pregnant women with CHB and assessed their potential value in predicting the decline of HBsAg after discontinuing treatment postpartum (13, 14). Chronic HBV infection is widely recognized as the result of viral-host immune interactions. Previous study indicated that pgRNA was correlated with Th1/Th2 immunity in non-pregnant patient with CHB (15). However, data on the correlations of HBcrAg and pgRNA, two novel virologic markers, with immunological parameters in pregnant women with CHB are still lacking.

Therefore, the study aimed to investigate the dynamics and correlations of cytokine profiles with HBcrAg and pgRNA in HBeAg-positive pregnant women with CHB undergoing prophylactic antiviral intervention.

## 2 Patients and methods

### 2.1 Study design and patients

We conducted a retrospective cohort study involving pregnant women with CHB enrolled at the outpatient clinic of the Department of Infection of the Second Affiliated Hospital of Chongqing Medical University between August 2015 and December 2019 with the following inclusion criteria: 1) Aged >18 years; 2) HBsAg positivity for > 6 months; 3) Pregnancy; 4) HBeAg positivity; 5) Received prophylactic antiviral intervention at 24-28 weeks of gestation; 6) Regular followed-up at 24-28 weeks of gestation, near birth, and within 3 months postpartum. Exclusion criteria: 1) Patients who initiated antiviral therapy pre-pregnancy; 2) Patients with comorbidities of other viral hepatitis (viral hepatitis A, C, D, and E) and human immunodeficiency virus (HIV) infection; 3) Patients with comorbidities of other liver diseases, such as liver fatty disease, autoimmune liver disease, etc.; 4) Patients with previous or existing decompensated cirrhosis and HCC; 5) Patients with other serious diseases in combination as assessed by the investigator; 6) Patients taking immunosuppressants or other medications that may affect the results of the study; 7) Patients with diseases assessed by the investigator as potentially affecting the results of the study. **Supplementary Figure S1** shows the flowchart of enrolled patients, 2 patients continued treatment postpartum and 26 patients discontinued treatment within 12 weeks after delivery based on the wishes of the patient and the decision of the physician.

This study confirms to the ethical guidelines of the 1975 Declaration of Helsinki, was approved by the Ethical Committee of the Second Affiliated Hospital of Chongqing Medical University and registered with the Chinese Clinical Trial Registry (ChiCTR2100054116). We obtained written informed consents from all participants.

### 2.2 Data collection and laboratory testing

Demographic information including age, labor history, duration of gestation, time of labor, as well as the treatment details including treatment regimen, duration of treatment, and time of discontinuation were collected from the study participants.

Clinical data including serum ALT, HBsAg, HBeAg, HBV DNA HBcrAg, pgRNA and cytokine profile were detected at 24-28 weeks of gestation (before treatment), near birth, and within 3 months postpartum (The parameters were detected within 1 month after treatment discontinuation). Serum ALT levels were assessed using Hitachi 7600-020, Tokyo, Japan (ULN: 40 U/L). HBsAg quantifications were performed by Roche COBAS HBsAgII-Q and the lower limit of detection (LLD) is 0.05 IU/ml. HBeAg quantifications were performed by Abbott GmbH & Co. KG and the LLD is 0.59 PEIU/mL. HBV DNA levels were detected by Roche Amplicor/COBAS TaqMan HBV test v2.0 (Roche Molecular Diagnostics, Pleasanton, CA, USA) and LLD is 100 IU/ml. Serum samples stored at -80°C were used for detecting the levels of novel virologic markers (HBcrAg and pgRNA) and cytokine profiles. HBcrAg levels were detected by Lumipulse^®^ G HBcrAg Fujirebiotech (Japan), with a LLD of 2.1 LogU/mL. PgRNA levels were detected by HBV RNA Quantitative Fluorescence Diagnostic Kit (Sansure Biotech Inc. China) with a LLD of 100 copies/mL.

Cytokines were detected by RayBiotech microarrays, including Th1-type cytokines: Interferon-Beta (IFN-β), Interferon-Gama (IFN-γ), Interleukin 12p40 (IL12p40), IL12p70, IL2, TNF-α (Tumor necrosis factor-α); Th2/Treg-type cytokines: IL4, IL5, IL6, IL10; Th17/Tfh-type cytokines: IL17, IL21, Inducible Synergistic Co-stimulation Molecules (ICOS); chemokines: Regulated upon Activation, Normal T cell Expressed and Secreted Expressed and presumably Secreted (RANTES), Macrophage Inflammatory Protein-1α (MIP-1α), Macrophage-Derived Chemokine (MDC), Monocyte Chemoattractant Protein-1 (MCP-1), Interferon γ Inducible Protein-10 (IP10), Growth-Related Oncogene (GRO), and cytokines regulating cell proliferation and differentiation: T cell Immunoglobulin domain and Mucin domain-3 (TIM-3), Transforming Growth Factor-β1 (TGF-β1), Programmed Death-1 (PD-1), IL23, IL18, IL15, Epidermal Growth Factor (EGF), Cytotoxic T Lymphocyte Associate protein-4 (CTLA4) and Cluster of Differentiation 40 Ligand (CD40L).

### 2.3 Outcome

The ALT flare is defined as > 2×ULN within 3 months postpartum, the ULN of ALT is 40 U/L. Viral rebound is defined as an increase in HBV DNA levels to more than 2000 IU/mL or an increase of more than 1 log10 from the lowest level (16).

### 2.4 Statistical analysis

Data are expressed as counts and percentages for categorical variables, and as mean (standard deviation) or median (range) for continuous variables. The student t-test or Mann-Whitney U-test was used for comparisons between continuous variables in different groups. Paired-samples t-tests or Mann-Whitney U tests were used for comparison between variables (ALT, HBsAg, HBV DNA, HBcrAg, pgRNA and cytokines) at different timepoints within groups. Discrimination Analysis (OPLS-DA) was used to compare the differential cytokines between the two groups, and cytokines with variables importance in the projection (VIP) values greater than 1 were displayed on the lollipop chart (17). Correlations between variables were analyzed by Spearman correlation analysis. Univariate and multivariate linear regression analyses were performed to identify the serum cytokines correlated with serum pgRNA, and cytokines with a *p* value less than 0.05 in univariate linear regression were enrolled in multivariate linear regression analysis. The correlation network diagram of variables was visualized by Cytoscape v.3.9.0 (18). Data were analyzed using SPSS version 24.0 (IBM Corp., Armonk, NY, USA) and R version 4.3.1 software. Two-sided *p* values were calculated for all tests and a *p* value < 0.05 was considered statistically significant.

## 3 Results

### 3.1 The baseline characteristics of HBeAg-positive pregnant women with CHB

**Table 1** shows the baseline characteristics of the enrolled pregnant women. The age of pregnant women ranged from 21-37 years old, 75.0% were younger than 30 years old. Most of the pregnant women had no parity history (82.1%), normal ALT levels (89.3%), and high viral loads (89.3%). Only one pregnant woman with CHB was diagnosed with compensated cirrhosis. Nine pregnant women received TDF prophylactic antiviral intervention at 24-28 weeks of gestation and 18 pregnant women received LdT prophylactic antiviral intervention, and 1 pregnant woman was switched to TDF treatment after 1 month of LdT treatment. A total of 92.9% pregnant women discontinued prophylactic antiviral intervention after delivery (26/28).

**Table 1:** The baseline characteristics of HBeAg-positive pregnant women with CHB.

Variable	
Parity *history*, n (%)	5 (17.9%)
Age < 30y, n (%)	21 (75.0%)
Normal ALT, n (%)	25 (89.3%)
HBV-DNA > 1.0E+06 IU/mL, n (%)	25 (89.3%)
Cirrhosis, n (%)	1 (3.6%)
TDF treatment, n (%)	9 (32.1%)
HBV-DNA, IU/mL	2.28E+07 (2.15E+05~1.47E+08)
qHBsAg, IU/mL	3.8E+04 (2.8E+03~1.08E+05)
PgRNA, pg/mL	1.5E+08 (3.25E+05~9.0E+08)
HBcrAg, pg/mL	8.40 (6.2~9.2)
ALT, U/L	25.6 (13~65)

ALT, alanine aminotransaminase; DNA, deoxyribonucleic acid; HBV, hepatitis B virus; HBcrAg, hepatitis B core related antigen; pgRNA, pre-genomic ribonucleic acid; qHBsAg, quantitative hepatitis B surface antigen; TDF, tenofovir disoproxil fumarate.

### 3.2 Overall dynamics of cytokine profiles and virologic markers in pregnant women undergoing prophylactic antiviral intervention

**Figure 1:**
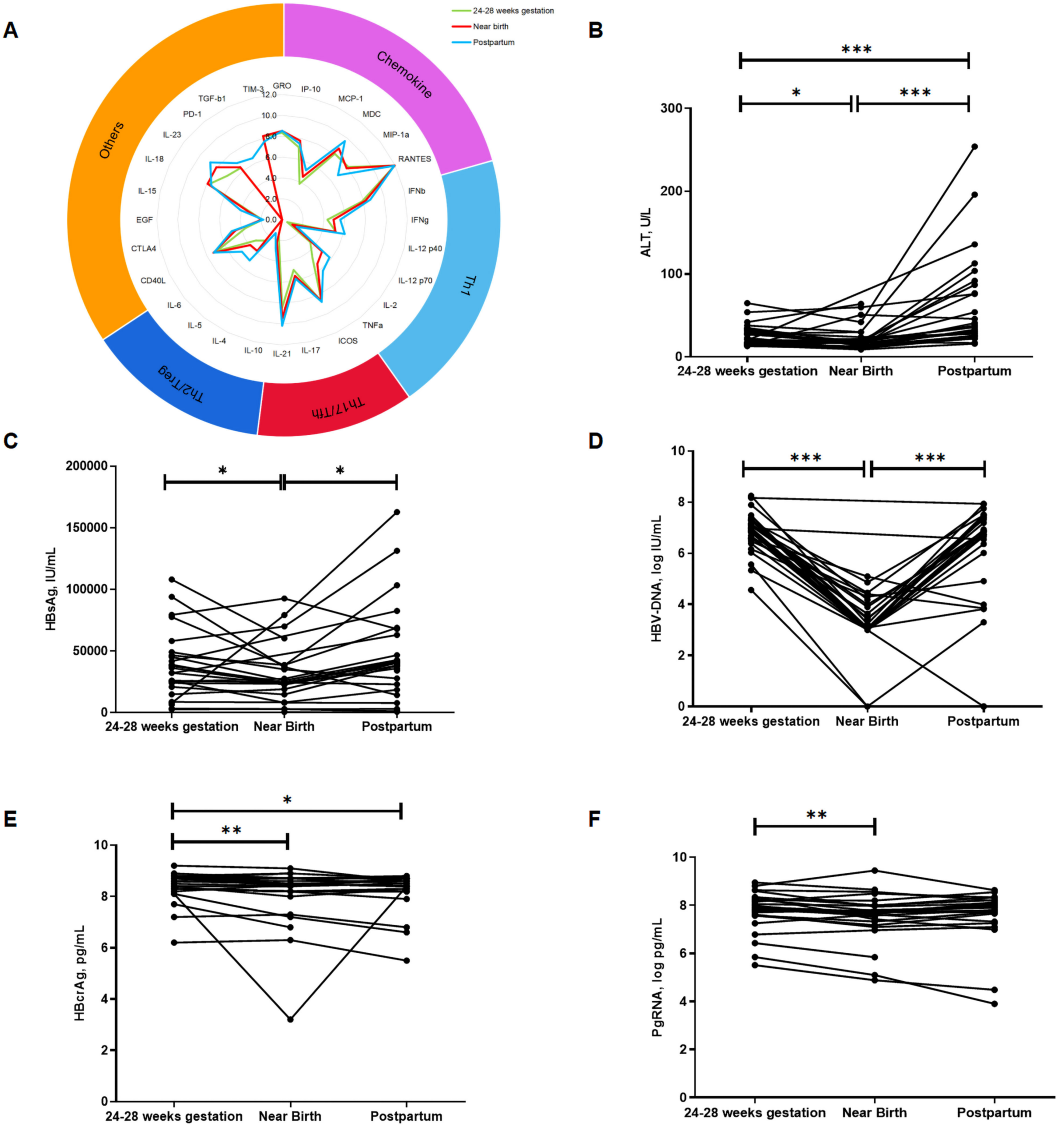
Overall dynamics in virologic markers and cytokine profiles during pregnancy and postpartum. The radiogram in **(A)** shows the dynamics in cytokine profiles at three time points, with different colors representing different time points. **(B–F)** shows the dynamics of ALT, HBsAg, HBV DNA, HBcrAg and pgRNA, respectively. Horizontal axis represents different time points, vertical axis represents ALT and different virological markers. p < 0.05 is marked by *, p < 0.01 is marked by ** and p < 0.001 is marked by ***.

We analyzed the overall dynamics of cytokine profiles and virological markers in HBeAg-positive pregnant women with CHB during pregnancy and postpartum (**Figure 1**). During pregnancy, levels of HBsAg, HBV DNA, HBcrAg, pgRNA, and ALT significantly decreased, whereas during the postpartum period, levels of HBsAg, HBV DNA, and ALT significantly increased. However, no significant changes were observed in levels of HBcrAg and pgRNA during the postpartum period. Cytokine profiles tended to increase during pregnancy, with statistically significant increases observed only for chemokines MCP-1 and IP-10. While during the postpartum period, multiple cytokines significantly increased, including chemokines (MCP-1 and IP-10), Th1-type cytokines (IFN-γ, IL12p40, IL12p70, IL2, and TNF-α), Th17/Tfh-type cytokines (IL17 and IL21), Th2-type cytokines (IL10, IL5, and IL6) and other cytokines regulating cell proliferation and differentiation (CTLA4, IL15, IL23, PD-1, and TGF-β1), suggesting an immune flare postpartum.

### 3.3 Cytokine profile was closely correlated with pgRNA

To further investigate viral-immune interactions, we analyzed the correlations of cytokine profiles with traditional virologic markers (HBsAg and HBV DNA), and novel virologic markers (HBcrAg and pgRNA).

**Figure 2:**
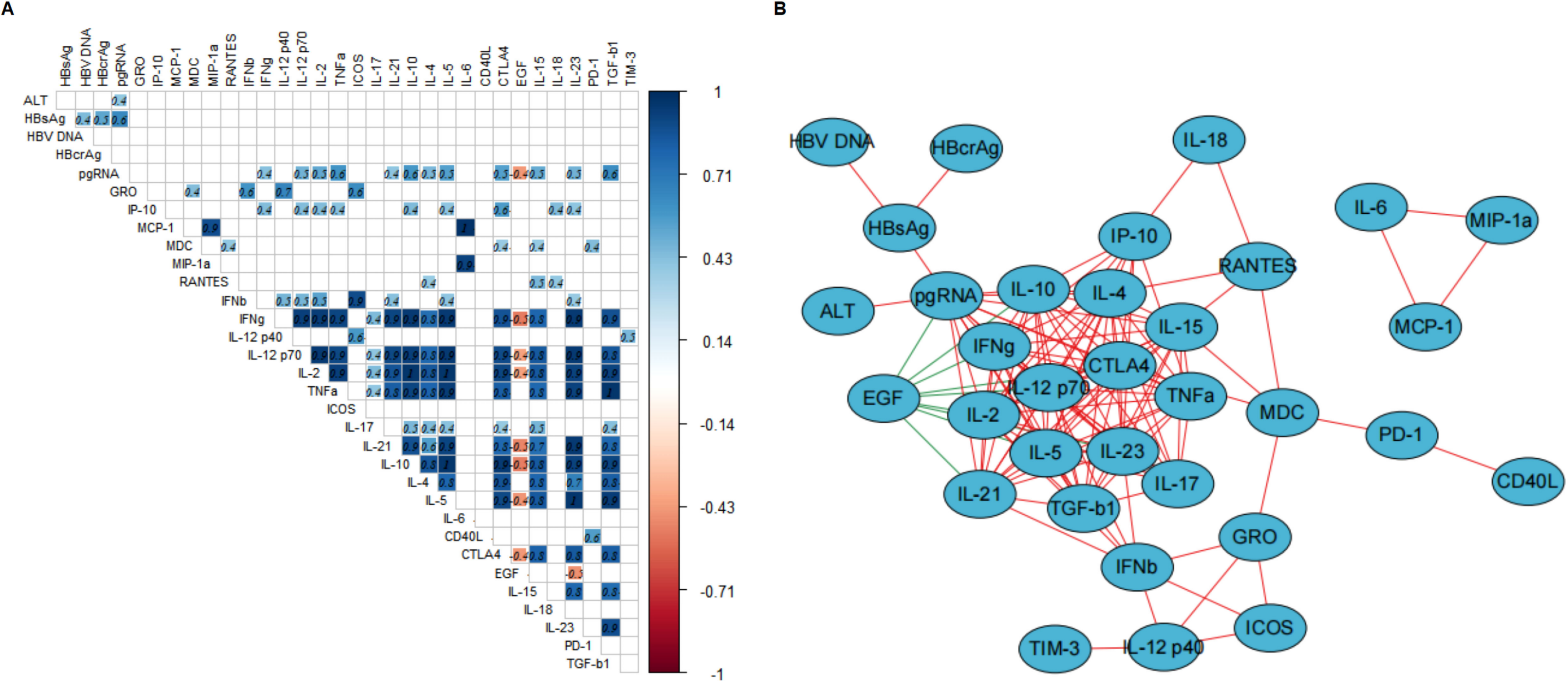
Correlation of virologic markers and cytokine profiles at time of 24-28 weeks of gestation. **(A, B)** describe the correlations between virologic markers and cytokine profiles at time of 24-28 weeks of gestation. **(A)** The correlation heatmap only shows the correlation coefficients with statistical significance (*p*-values less than 0.05), the number in each square represents the correlation coefficient between two variables. The blue squares represent positive correlations and red squares represent negative correlations, and the darker the color, the stronger the correlation between two variables. **(B)** The correlation network diagram (visualized by Cytoscape) shows the correlation between virologic markers and cytokines and the variables in the center of the diagram indicating more complicated correlation with other variables. The red line and green line between the variables represent positive correlation, and negative correlation, respectively. The thickness of the lines represents the strength of the correlation, the thicker the line, the stronger the correlation.

Before prophylactic antiviral intervention, HBsAg was significantly positively correlated with HBV-DNA, HBcrAg, and pgRNA. PgRNA was moderately (correlation coefficient between 0.4 and 0.6) positively correlated with multiple cytokines (**Figure 2A**), including Th1-type cytokines (IFN-γ, IL12p70, IL2, and TNF-α), Th17-type cytokines (IL21), Th2-type cytokines (IL10, IL4, and IL5), and cytokines regulating cell proliferation and differentiation (CTLA4, IL15, IL23, and TGF-β1) and was moderately negatively correlated with EGF (correlation coefficient = -0.4). While ALT, HBV-DNA, HBsAg and HBcrAg showed no significant correlation with cytokines. Furthermore, a network diagram based on the correlation coefficients between the variables (virologic markers and cytokines) revealed that pgRNA correlated more closely with the cytokine profile (**Figure 2B**). We further explored the biological processes, signaling pathways, and cytokine interactions involved in cytokines closely correlated with pgRNA (**Supplementary Figure S2**). To further confirm the correlations between cytokines and pgRNA, we performed the univariate and multivariate linear regression analyses. The results from the univariate linear regression analysis are consistent with the results of the Spearman correlation analysis. And multivariate linear regression analysis indicated that cytokines IFN-γ and IL4 were independently and negatively correlated with pgRNA, and IL10 was independently and positively correlated with pgRNA (**Supplementary Table S1**).

**Figure 3:**
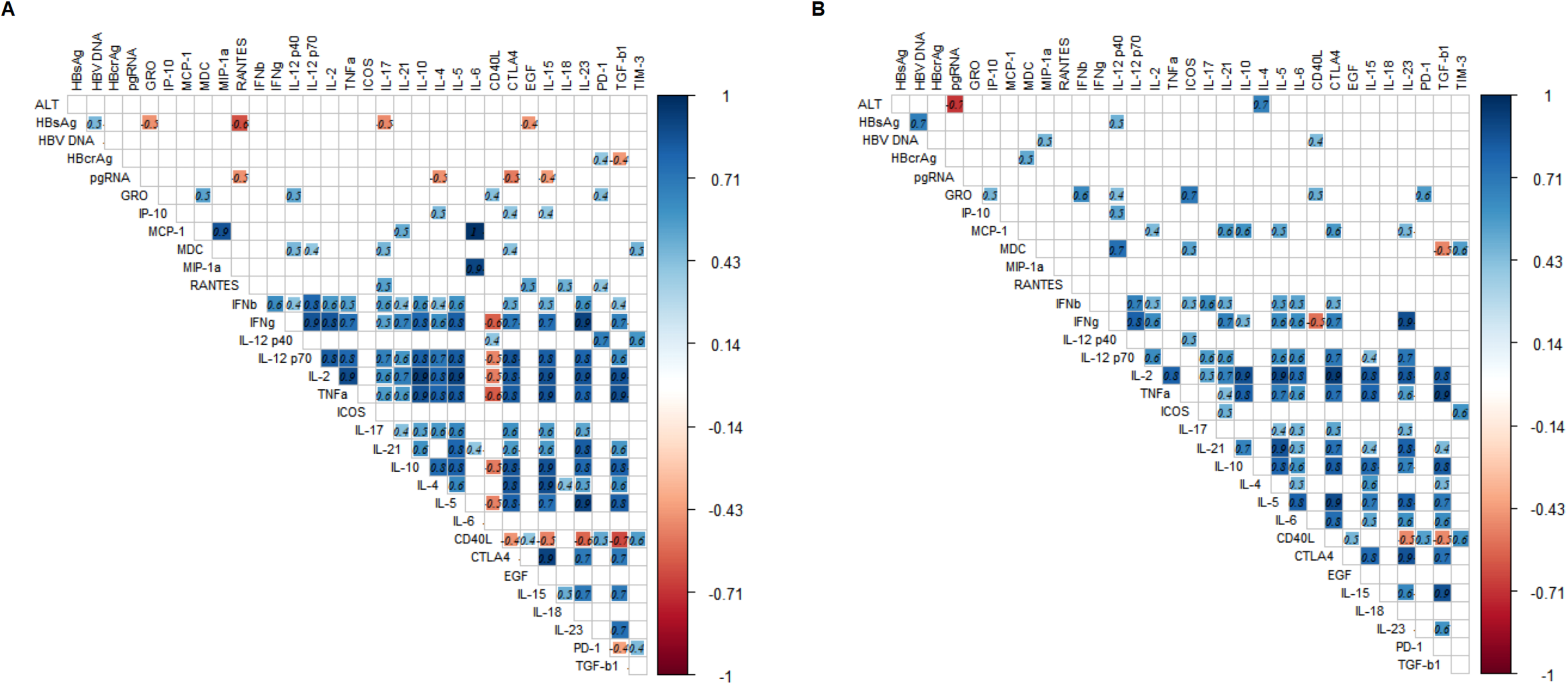
Heatmap of correlation between changes in virologic markers and cytokine profiles during pregnancy and postpartum. **(A, B)** describe the correlation between changes in parameters during pregnancy and patients experienced viral rebound postpartum, respectively. The correlation heatmap only shows the correlation coefficients with statistical significance (*p*-values less than 0.05), the number in each square represents the correlation coefficient between two variables. The blue squares represent positive correlations and red squares represent negative correlations, and the darker the color, the stronger the correlation between variables.

We further investigated the correlations between the changes in cytokine profiles and the changes in HBcrAg and pgRNA after prophylactic antiviral intervention. Changes in HBsAg were significantly positively correlated with changes in HBV DNA and negatively correlated with changes in cytokines GRO, RANTES, IL17, and EGF. Changes in HBcrAg were significantly positively correlated with changes in PD-1 and negatively correlated with changes in TGF-β1. Additionally, changes in pgRNA were significantly negatively correlated with changes in cytokines RANTES, IL4, CTLA4, and IL15. However, changes in ALT and HBV DNA were not significantly correlated with changes in cytokines. The above results indicated that cytokine profiles during pregnancy in HBeAg-positive pregnant women with CHB undergoing prophylactic antiviral intervention closely correlated with virologic changes (**Figure 3A**).

### 3.4 Changes in virologic markers closely correlated with cytokine profiles in postpartum women who experienced virologic rebound

**Figure 4:**
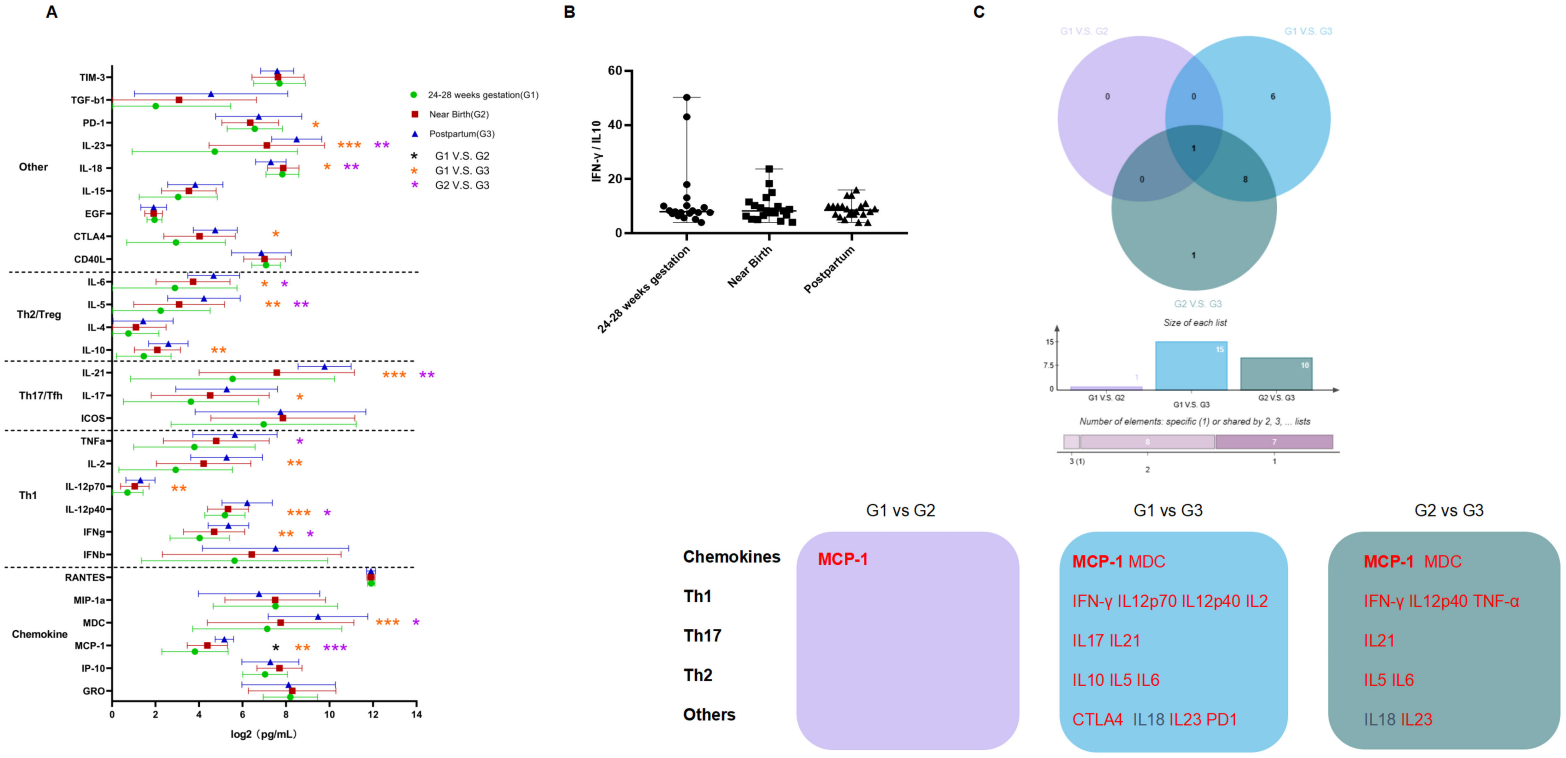
Dynamics in cytokine profiles and Th1/Th2 ratio in patients who experienced viral rebound. **(A)** The horizontal axis represents the levels of cytokines (log2 transformed), the vertical axis represents different cytokines; Green, red and blue represents 24-28 weeks of gestation, near birth and postpartum, respectively; Black * represents comparisons between 24-28 weeks of gestation and near birth; orange * represents comparisons between 24-28 weeks of gestation and postpartum, and purple * represents comparisons between near birth and postpartum. **(B)** The horizontal axis represents different time points, and the vertical axis represents the ratio of Th1/Th2-type cytokines. **(C)** Purple represents the comparison between 24-28 weeks of gestation and near birth, blue represents the comparison between 24-28 weeks of gestation and postpartum, and green represents the comparison between near birth and postpartum. Red and black fonts represent higher and lower level of cytokines at the latter timepoint compared to the previous timepoint, respectively. Bolded font represents cytokines that changed in all three comparisons. P < 0.05 is marked by *, P < 0.01 is marked by **, and P < 0.001 is marked by ***.

Twenty-six postpartum women discontinued treatment, of whom 24 experienced virologic rebound. We found that levels of ALT, HBsAg, HBV DNA and pgRNA after discontinuing treatment postpartum were significantly increased compared to levels at the time of near birth, except for HBcrAg, which showed no significant increase. In addition, levels of ALT and HBsAg were even increased over the levels before prophylactic antiviral intervention, while levels of HBV DNA and pgRNA were returned to the levels before prophylactic antiviral intervention (**Supplementary Figure S3**). Most cytokines were significantly increased after discontinuing treatment postpartum (**Figure 4A**), including cytokines regulating cell proliferation and differentiation (PD-1, IL23, IL18, and CTLA4), Th2/Treg-type cytokines (IL5, IL6, and IL10), Th17/Tfh-type cytokines (IL17 and IL21), Th1-type cytokines (IFN-γ, IL12p40, IL12p70, IL2, and TNF-α) and chemokines (MCP-1 and MDC). Further analysis showed no significant change in the Th1-type/Th2-type cytokine ratio (**Figure 4B**). The Wayne diagram indicated the cytokine that were significantly changed during pregnancy and after discontinuing treatment postpartum (**Figure 4C**).

We proposed to explore the role of cytokine profiles in viral rebound. Therefore, we analyzed the correlation between the changes of cytokine profiles and virologic markers. The change in ALT was positively correlated with the change in IL4 and negatively correlated with the change in pgRNA. The change in HBsAg was positively correlated with the changes in HBV DNA and IL12p40. The change in HBV-DNA was positively correlated with the changes in MIP-1α and CD40L and the change in HBcrAg was positively correlated with the change in MDC, whereas the change in pgRNA was not significant correlated with the changes in cytokines (**Figure 3B**).

### 4.5 Cytokine profiles varied in different populations of pregnant women with CHB

**Figure 5:**
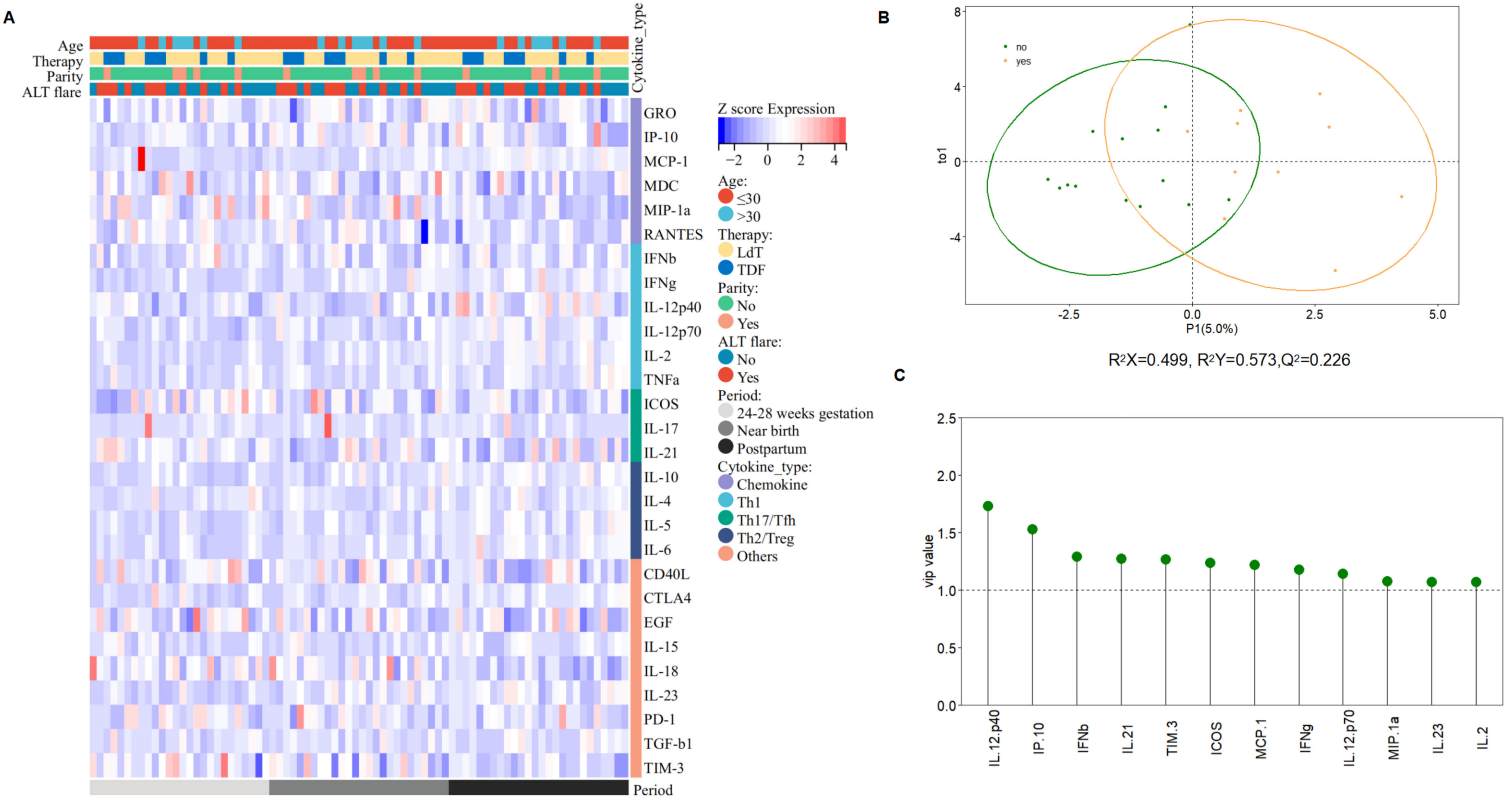
Subgroup analysis of cytokine profile differences. **(A)** The heatmap visualized by Sangerbox (31) describes cytokine profile difference regarding age, parity history, treatment regimen, ALT flare at three time points. Red represents high levels of cytokines, the darker the color, the higher the level, and blue represents low levels of cytokines, the darker the color, the lower the level. **(B)** The score plot analyzed by OPLS-DA between patients developed ALT flare and who did not (R^2^X=0.499, R^2^Ycum=0.573, Q^2^ = 0.226). **(C)** VIP (variables important in the projection) values, explaining the extent to which a variable contributes to the projection, normally used as a criterion to identify variables important to the discrimination method developed.

To further confirm the differences in cytokine profiles and dynamics in pregnant women with different characteristics, we performed subgroup analyses to characterize differences in cytokine profiles and dynamics during pregnancy and postpartum after treatment discontinuation in pregnant women with CHB, based on age, parity history, and antiviral regimens (**Figure 5**). There were no significant differences in cytokine profiles among pregnant women regarding age, except that a higher level of MIP-1α was found in patients older than 30 years old at postpartum period compared to those younger than 30 years old. Cytokine increases were more pronounced in pregnant women younger than 30 years old during pregnancy and postpartum period compared to those older than 30 years old. Pregnant women with CHB with parity history had lower MIP-1α levels at 24-28 weeks of gestation, but higher MIP-1α levels after discontinuing treatment postpartum compared to those without parity history. Cytokine profiles changes were more pronounced after discontinuing treatment postpartum in pregnant women with CHB without parity history compared to those with parity history. In addition, we found significant differences in cytokine profiles between pregnant women received different antiviral regimens. The effect of TDF administration on cytokine profile during pregnancy in pregnant women with CHB was more pronounced than that of LdT administration, showing significant increases in the levels of MCP-1, IFN-β, IFN-γ, IL2, IL21, and IL23. Moreover, the two antiviral drugs had different effect on the dynamics of cytokine profiles after discontinuing treatment postpartum.

### 3.6 Postpartum ALT flare exhibited immune activation

Previous studies indicated that postpartum ALT flare may be closely related to immune changes. In this study, the incidence of postpartum ALT flare after discontinuing treatment was 42.3% (11/26). We compared the difference and changes of virologic markers and cytokine profiles in pregnant women regarding postpartum ALT flare in order to explore the virologic markers and cytokines that may be correlated with the development of postpartum ALT flare (**Supplementary Figure S4**).

Levels of HBcrAg and pgRNA significantly decreased during pregnancy in pregnant women who did not develop ALT flare, while levels of HBsAg significantly increased after discontinuing treatment postpartum in pregnant women who developed ALT flare. The dynamics of HBV DNA were consistent between the two groups, showing a significant decrease during pregnancy and a significant increase after discontinuing treatment postpartum, returning to levels at 24-28 weeks of gestation. Levels of ALT were significantly increased after discontinuing treatment postpartum regardless of the development of ALT flare, while a higher level of ALT was observed in patients who developed postpartum ALT flare at postpartum period compared to those who did not (**Supplementary Figure S4A**).

Cytokine profiles showed no significant difference between the two groups at 24-28 weeks of gestation and near birth, whereas higher levels of IFN-γ, IL2, IL21, and IL10 were observed in patients who developed ALT flare after discontinuing treatment postpartum (**Supplementary Figure S4B**). Moreover, we found that more significant increases of cytokine profiles in patients who developed ALT flare after discontinuing treatment postpartum compared to those who did not (**Supplementary Figure S4C**). We further analyzed the differences in cytokine profiles between the two groups by OPLS-DA to identify the cytokines with VIP values ≥1, which were ranked based on the value, and finally screened the key cytokines that might be associated with the development of postpartum ALT flare. Data in **Figure 5** showed that IL12p40 was the most crucial differential cytokine, followed by cytokines IP-10, IFN-β, IL21, TIM-3, ICOS, MCP-1, IFN-γ, IL12p70, MIP-1α, IL23, and IL2.

## 4 Discussion

This study is the first to comprehensively characterize the dynamics of cytokine profile, as well as HBcrAg and pgRNA profiles, and their correlations in HBeAg-positive pregnant women with CHB undergoing prophylactic antiviral intervention. We found a closer correlation between pgRNA and cytokine profiles before prophylactic antiviral intervention. After prophylactic antiviral intervention, the levels of virologic markers decreased while the immune status was relatively stable during pregnancy except for the increases of chemokines MCP-1 and IP-10, and the changes of virologic markers were closely correlated with cytokines. After postpartum treatment discontinuation, immune activation occurred, and additionally, most participants experienced virologic rebound and 42.3% experienced ALT flare. Changes in virologic markers were closely correlated with cytokines, and postpartum ALT flare may be the result of the interactions between Th1-type cytokines and chemokines, with IL12p40 potentially being the most crucial cytokine. Therefore, our findings improve the understanding of pregnant women with CHB undergoing prophylactic antiviral intervention in terms of viral-immune interactions.

Previous studies have demonstrated that maternal immune response during pregnancy, regulated by estrogen and progesterone, is shifted toward Th2 and Treg and manifests as immunosuppression. In contrast, hormone levels drop significantly after delivery, causing the postnatal immune response to shift toward Th1 and Th17, manifesting immune activation (19, 20). Studies related to cytokine changes from late pregnancy to near delivery in healthy pregnancies were limited (19, 21). Regarding cytokine changes in the postpartum period, it’s reported that both pro-inflammatory (IFN-γ and IL2) and anti-inflammatory cytokines (IL4 and IL10) levels were increased postpartum, but the increase in pro-inflammatory cytokines was more pronounced in healthy pregnancies (21, 22). There is a lack of immunological studies on pregnant women with CHB undergoing prophylactic antiviral intervention (23–25). The present study first found that HBeAg-positive pregnant women with CHB undergoing prophylactic antiviral intervention had insignificant changes in cytokine profiles during late pregnancy, with only increases in chemokines, whereas levels of various cytokines increased significantly after discontinuing treatment postpartum, with an equal degree of Th1-type and Th2-type immune activation. Dynamics in Th1/Th2 ratio in pregnant women with CHB may differ from healthy pregnancies, which are characterized by elevated Th1/Th2 ratios postpartum (21). Therefore, the immune response may be more complex under prophylactic antiviral intervention, delivery and treatment discontinuation. In addition, our study found that cytokine profiles differed by age and delivery history, and that antiviral drugs, indicating the cytokine profiles were different in different host factors and antiviral drugs. And future well-designed prospective studies with large sample are needed to clarify the impact of these factors on dynamics of cytokine profiles in pregnant women with CHB.

There are fewer studies on the correlation between novel virologic markers and immunity, especially in pregnant women with CHB. A previous study suggested that serum pgRNA levels were negatively correlated with Th1-type cytokines and positively correlated with Th2-type cytokines in non-pregnant CHB patients (15). In the present study, we found for the first time that in HBeAg-positive pregnant women with CHB, pgRNA were more closely correlated with cytokines than HBcrAg and traditional markers (HBV DNA and HBsAg) at 24-28 weeks of gestation, and that pgRNA was positively correlated with Th1 cytokines, Th2 cytokines, and Th17 cytokines, and negatively correlated with EGF. Changes of virologic markers were closely correlated with cytokine. Multivariate linear regression analysis indicated that cytokines IFN-γ and IL4 were independently and negatively correlated with pgRNA, and IL10 was independently and positively correlated with pgRNA. The viral-immune interactions are more complicated in pregnant women with CHB and different from non-pregnant patients. PgRNA can be recognized by the retinoic acid-inducible gene-I (RIG-I), a pattern recognition receptor of the host, activating the immune response and promoting the expression of IFN-γ (26). In turn, the immune status may influence the HBV replication (27, 28). Future studies could focus on how cytokine-related signaling pathways and upstream and downstream key immune cells are involved in HBV synthesis and transcription in pregnant women with CHB.

Immune reactivation is thought to underlie the development of postpartum ALT flare (24). Previous studies have suggested significant activation of CD4+ and CD8 +T cells in patients with postpartum ALT flare (7, 29). Recently, Liu Y et al. reported that the postpartum period in pregnant women with CHB may be an opportunity to achieve clinical cure, as evidenced by the high rate of HBsAg loss after interferon combined with oral antiviral treatment (30). Therefore, research on the mechanism underlying postpartum ALT flare may provide valuable insights into cure of CHB. Cytokines are secreted by various immune cells, which are the executors of the immune response, thus analysis of cytokine profiles contributes to the understanding of the overall immune response in patients developed postpartum ALT flare. Since cytokines correlate with each other, we used the OPLS-DA method to identify the differences in cytokine profiles between patients who developed postpartum ALT flare and those who did not. Our results suggest that postpartum ALT flare may be the result of the interactions between Th1-type cytokines and chemokines, and IL12p40 may be the most crucial cytokine, which may help to identify key targets for future studies on the immune mechanisms underlying ALT flare.

Our study adds to the knowledge of immunology and its correlation with virology in HBeAg-positive pregnant women with CHB. There are also several limitations to this study. First, this study was a retrospective study with a small sample size, maternal baseline characteristics, viral characteristics, antiviral treatment, and sampling time points may influence the results. However, we included pregnant women with similar age, high viral load and receiving first-line antiviral regimens to minimize the bias. And paired-samples Mann-Whitney tests were used to analyze the dynamics of variables to improve the test efficacy. Nonetheless, future well-designed prospective cohorts with large samples are needed to further validate our findings and clarify the impact of confounding factors. Second, only 2 subjects chose to continue treatment postpartum, which limits our ability to determine whether the postpartum immune activation was caused by delivery or discontinuation of treatment. Finally, we identified cytokines that were closely correlated to the occurrence of postpartum ALT flare, which needs to be further validated by basic experiments.

## 5 Conclusions

PgRNA was more closely correlated to cytokine profiles, and postpartum ALT flare may be the result of the interaction between Th1-type cytokines and chemokines.

## Data Availability

The raw data supporting the conclusions of this article will be made available by the authors, without undue reservation.
