# Supplementary material for: PgRNA closely correlates to cytokine profile in HBeAg-positive pregnant women undergoing prophylactic antiviral intervention
Source: Front Immunol. 2024 Dec 11;15:1511855. doi: 10.3389/fimmu.2024.1511855 (PMC11668345; doi:10.3389/fimmu.2024.1511855)
Supplement: Supplementary file 1 [file Table1.docx]

**PgRNA closely correlates to cytokine profile in HBeAg-positive pregnant women undergoing prophylactic antiviral intervention.**

Qiao Tang^3^, Chunrui Wang^3^, Hu Li^3^, Zhiwei Chen^3^, Xiaoqing Liu^3^, Yunling Xue^3^, Yue Qiu^3^, Yi Zeng^3^, Peng Hu^1, 2*^.

Supplementary table

Table S1 Univariate and multivariate linear regression analyses of serum cytokines correlated with serum pgRNA.

| Cytokine | Univariate | | | Multivariate | | |
| --- | --- | --- | --- | --- | --- | --- |
|  | Adjusted B | 95% Confidence Interval (CI) | *P* value | Adjusted B | 95% CI | *P*value |
| GRO | -0.054 | -383200.050~290240.883 | 0.779 |  |  |  |
| IP10 | 0.251 | -255496.989~1232572.043 | 0.189 |  |  |  |
| MCP1 | 0.046 | -1737392.061~2193303.164 | 0.814 |  |  |  |
| MDC | 0.276 | -52889.315~333900.634 | 0.148 |  |  |  |
| MIP-1α | -0.137 | -67367.617~32362.774 | 0.478 |  |  |  |
| RANTES | -0.019 | -203383.471~184763.346 | 0.922 |  |  |  |
| IFN-β | 0.106 | -111796.081~194205.784 | 0.585 |  |  |  |
| IFN-γ | 0.407 | 424472.868~7116414.874 | 0.029 | -1.491 | -23655085.840~-4010289.501 | 0.009 |
| IL12p40 | -0.173 | -4334342.307~1666260.102 | 0.370 |  |  |  |
| IL12p70 | 0.472 | 23711506.620~156589642.200 | 0.010 | 0.843 | -63011778.290~384985023.000 | 0.146 |
| IL2 | 0.507 | 917935.007~4659569.520 | 0.005 | -0.209 | -7886157.647~5584091.994 | 0.721 |
| TNF-α | 0.597 | 718318.604~2342922.661 | 0.001 | 0.646 | -1518334.623~4832369.676 | 0.284 |
| ICOS | -0.037 | -66155.542~54902.413 | 0.850 |  |  |  |
| IL17 | 0.186 | -540069.182~1529467.275 | 0.335 |  |  |  |
| IL21 | 0.408 | 17738.473~287842.998 | 0.028 | -0.498 | -500987.750~128246.283 | 0.226 |
| IL10 | 0.573 | 12905108.610~46486050.250 | 0.001 | 2.075 | 22063935.940~193213277.300 | 0.017 |
| IL4 | 0.457 | 4438871.608~33902669.450 | 0.013 | -1.213 | -86779054.780~-15009890.900 | 0.009 |
| IL5 | 0.543 | 2423375.594~10039121.580 | 0.002 | -1.233 | -33509488.190~5187021.668 | 0.140 |
| IL6 | -0.065 | -311553.569~223031.771 | 0.737 |  |  |  |
| CD40L | -0.118 | -1468578.691~788556.383 | 0.542 |  |  |  |
| CTLA4 | 0.544 | 1667200.911~6862033.333 | 0.002 | 0.306 | -4947549.696~9738112.556 | 0.498 |
| EGF | -0.407 | -170105884.700~-10162463.780 | 0.029 | -0.253 | -124584994.600~12295046.750 | 0.101 |
| IL15 | 0.508 | 2325854.003~11777394.440 | 0.005 | 0.414 | -1511808.761~13000360.290 | 0.112 |
| IL18 | -0.016 | -641286.398~590414.904 | 0.933 |  |  |  |
| IL23 | 0.470 | 95235.671~643924.000 | 0.010 | -0.453 | -1472303.982~759793.047 | 0.507 |
| PD1 | -0.040 | -477035.647~388384.788 | 0.835 |  |  |  |
| TGF-β1 | 0.630 | 471517.09~1368091.889 | <0.001 | 1.165 | -283777.838~3689391.263 | 0.088 |
| TIM-3 | 0.006 | -467124.76~481930.859 | 0.975 |  |  |  |

Note: Cytokines with a *p* value less than 0.05 in univariate linear regression were enrolled in multivariate linear regression analysis. The large confidence intervals may be caused by the small sample size and the large variation in the concentration of different cytokines.

**Supplementary figures**

**
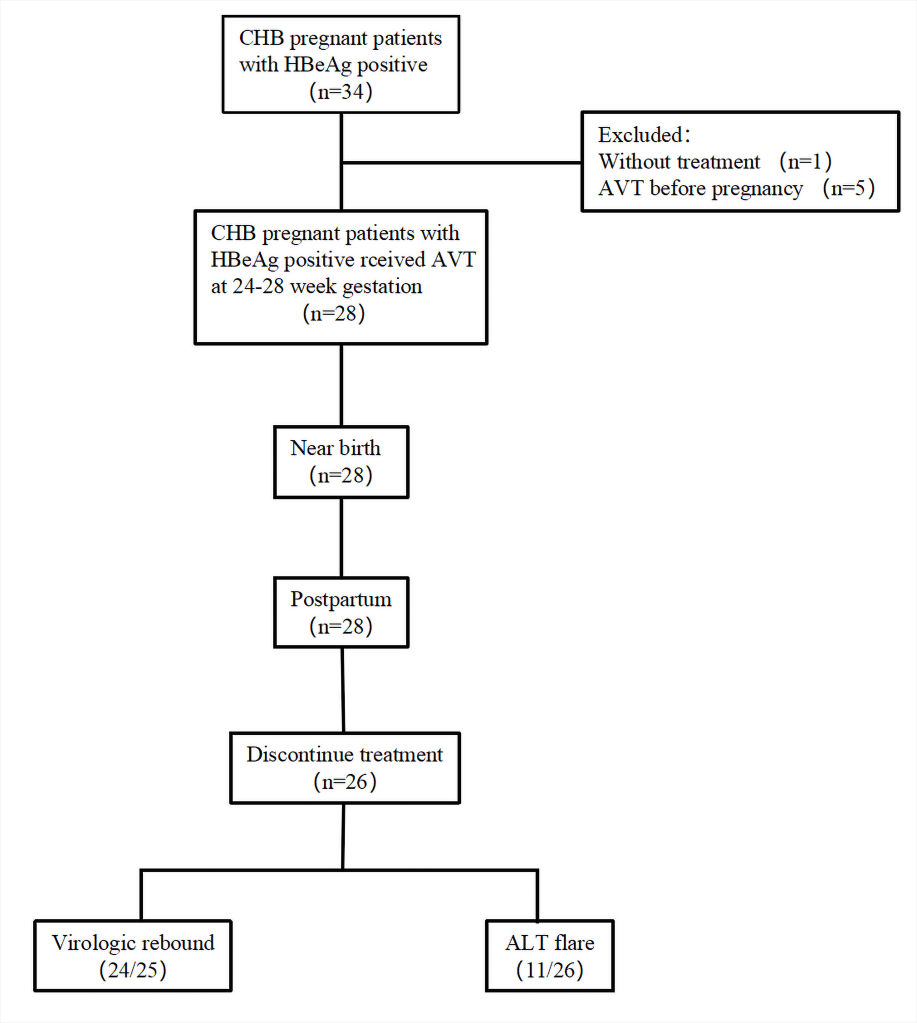
**

**Fig. S1 The flowchart of enrolled patients.**

**
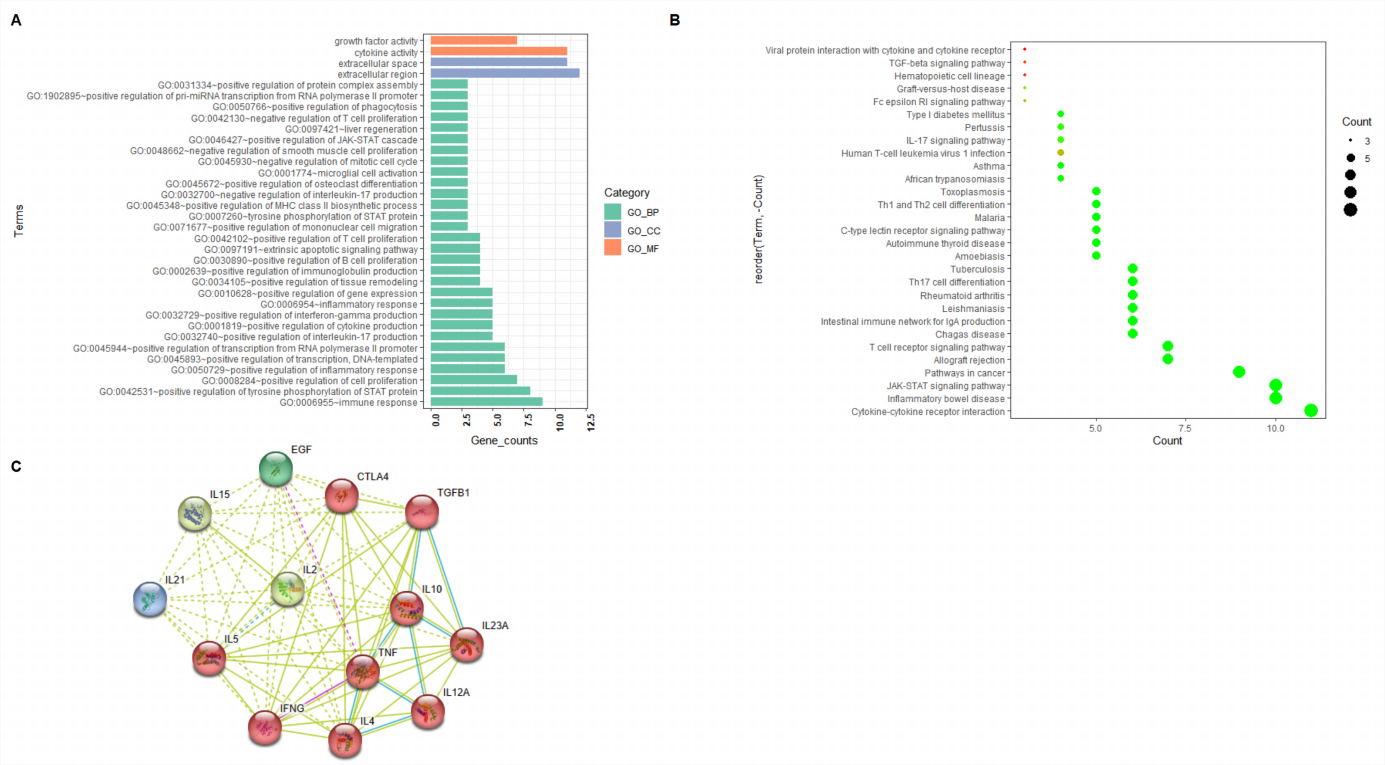
**

**Fig. S2 GO, KEGG and PPI analysis of cytokines with correlations with PgRNA. (A)** The horizontal axis represents the number of genes, in the vertical axis, the green, blue and orange bars represent biological process, cellular component and molecular function, respectively. **(B)** The horizontal axis represents the number of genes, vertical axis represents signaling pathways, and the color of bubbles represents the size of P-value. **(C)** Visualized by STRING database, clustered by K-means, and different colors represent different clusters.


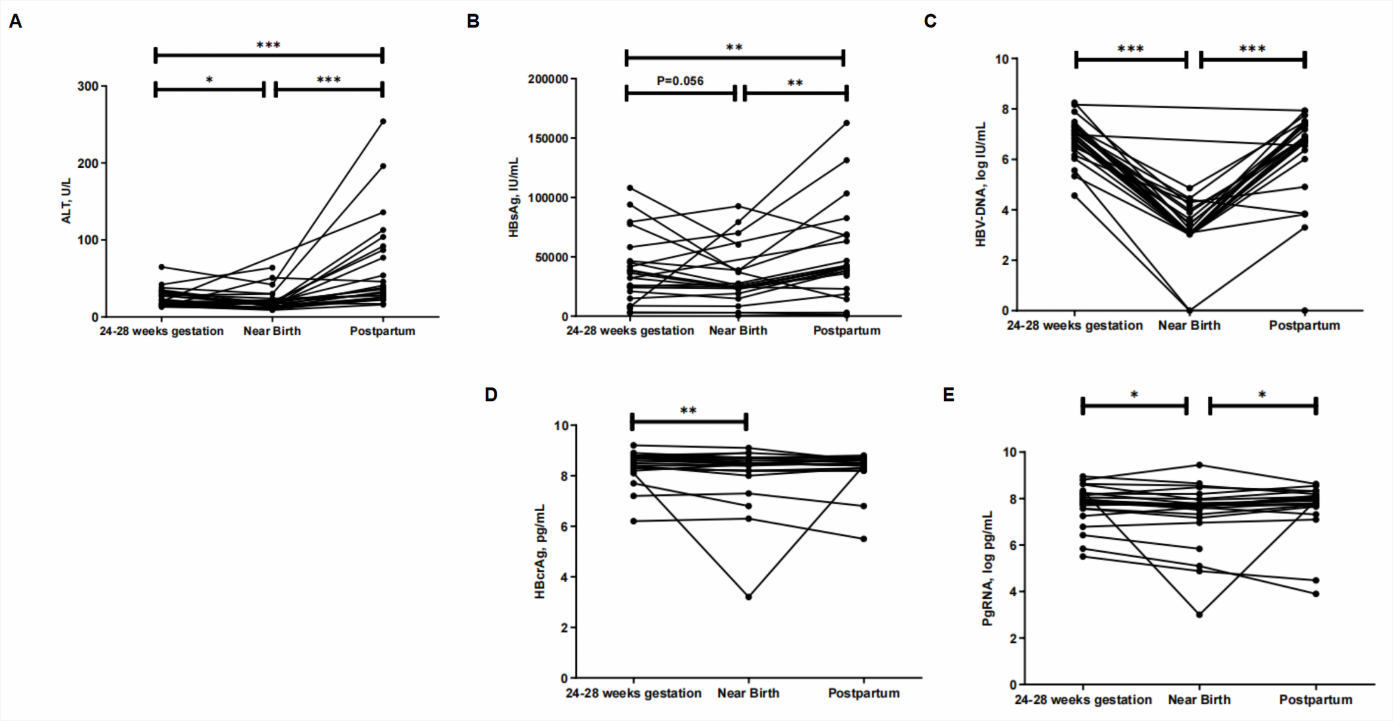


**Fig. S3 Dynamics of virologic markers and ALT in patients with virological rebound after treatment discontinuation postpartum.** The horizontal axis represents different time points and the vertical axis represents the levels of ALT and virologic markers. p < 0.05 is marked by *, p < 0.01 by **, and p < 0.001 by ***.


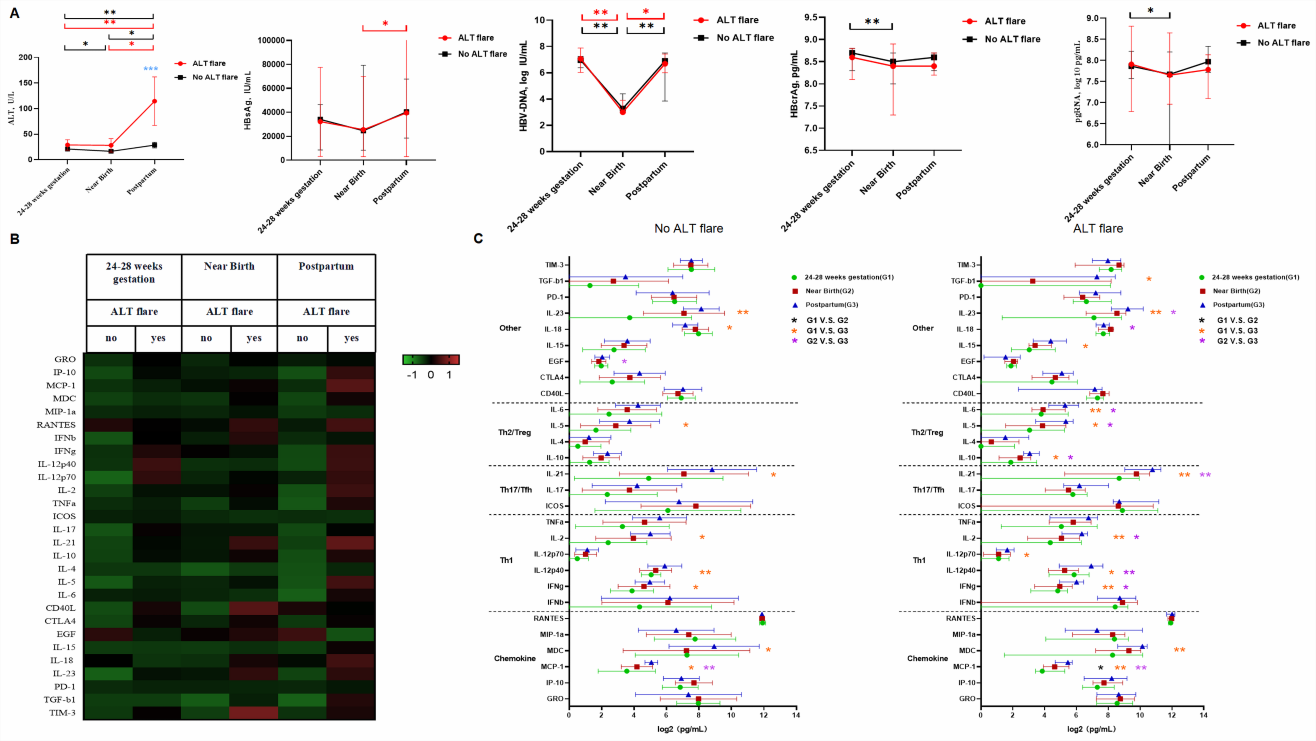


**Fig. S4** **Dynamics of virological markers and cytokine profile in postpartum women grouped by ALT flare.** **(A)** The horizontal axis represents different time points and the vertical axis represents the levels of ALT and virologic markers. **(B)** Comparison of cytokine profiles in pregnant with and without ALT flare. **(C)** Dynamics of cytokine profile in patients with and without ALT flare. Green, red and blue for 24-28 weeks of gestation, near birth; and postpartum; Black *represents comparisons between 24-28 weeks of gestation and near birth; orange * represents comparisons between 24-28 weeks of gestation and postpartum, and purple * represents comparisons between near birth and postpartum. P < 0.05 is marked with *, P < 0.01 with **, and P < 0.001 with ***.
